# Pediatric respiratory syncytial virus infections associated with hospital airborne viral genetic load detection

**DOI:** 10.1017/ice.2025.10372

**Published:** 2026-03

**Authors:** Carlos Alfaro-Perez, Rosa de Llanos, Luis Alfredo Herrero Cucó, Juana Maria Delgado-Saborit

**Affiliations:** 1 Department of Medicine, Faculty of Health Sciences. Universitat Jaume I, Castellón de la Plana, Spain; 2 Hospital General Universitario Castellón, Castelló de la Plana, Spain; 3 Epidemiology and Environmental Health Joint Research Unit, Foundation for the Promotion of Health and Biomedical Research in the Valencian Region, FISABIO-Public Health, FISABIO–Universitat Jaume I–Universitat de València, Valencia, Spain

## Abstract

**Objectives:**

This study investigates the potential aerosol transmission of respiratory syncytial virus (RSV), a major cause of viral pneumonia and bronchiolitis in young children.

**Methods:**

Two hundred samples were collected in a long-term environmental surveillance program from January 2022 until January 2023. Samples were collected in a pediatric emergency corridor. The analyses were performed using reverse transcription-quantitative polymerase chain reaction (RT-qPCR) targeting the RSV matrix gene. Information on the daily number of emergencies related with pediatric RSV infections was provided by the hospital.

**Results:**

Aerosol samples collected from a pediatric hospital corridor revealed detectable RSV RNA, particularly during peak infection seasons. RSV RNA was detected in 35 of 200 aerosol samples with a median concentration (interquartile range) of 1.8 (4.1) gc/m^3^. During the month of the peak season of RSV infections (November), RSV RNA was detected in 95% of the aerosol samples. Correlation analysis suggests a link between pediatric RSV cases and airborne RSV RNA concentration.

**Conclusions:**

RSV RNA has been detected in aerosols in a healthcare setting, particularly during peak infection periods. This does not constitute evidence of transmission of the RSV via aerosols. However, the observed correlation with pediatric RSV cases suggests that further research on viral viability and infectivity from RSV detected in aerosols should be conducted. It also shows the potential of characterizing RSV RNA in aerosols for environmental surveillance purposes.

## Introduction

Respiratory syncytial virus (RSV) is a negative-stranded RNA virus belonging to the *Pneumoviridae* family.^1^ It is widely distributed globally and is one of the leading causes of hospitalization for acute lower respiratory tract infections, especially in infants, young children and older adults.^2^ RSV infection is among the leading causes of mortality in infants under one year of age.^3^ Globally, RSV is associated with 3.2 million hospitalizations in children under 5 years old and between 94 600 and 149 400 deaths, of which approximately half occur in infants under 6 months old, the majority in developing countries.^4,5^

Recently, some vaccines and protective drugs have become available to lower the health burden associated with RSV infections.^5–7^ However, these vaccines might not be readily accessible for a large proportion of the population worldwide. Thus, protocols aimed at reducing viral transmission, might still be the first prophylactic measure for this population.

Recent evidence from other viruses, such as SARS-CoV-2, for which an airborne transmission has been acknowledged,^8^ calls to question whether other respiratory viruses could also be transmitted via aerosols. Two routes of RSV transmission have been mainly described in the literature. First, by large droplets (10–100 µm) propelled short distances (≤0.9 m) by coughing, sneezing, or even exhalation; and secondly, by contamination of environmental surfaces with infectious secretions and subsequently by self-inoculation.^9–13^ The classic experimental study by Hall and Douglas (1981), in which volunteers were exposed to infected infants through three different modes (“cuddlers,” “touchers,” and “sitters”), further supported these routes. While cuddlers (close contact) and touchers (self-inoculation via contaminated surfaces) became infected, sitters who remained more than 6 feet away—thus only exposed to potential aerosols—did not acquire infection, suggesting that aerosol transmission was not a major route under those conditions.^14^ In line, most infection control practices and strategies against RSV center on the prevention of transmission through contact with contaminated surfaces or direct contact.^15–17^ Notwithstanding, in 2022, the European Centre for Disease Prevention and Control, following lessons learned from the COVID-19 pandemic, included ventilation of indoor spaces as one of the good hygienic measures to reduce RSV transmission.^18^ Despite the traditional view that RSV was not transmitted via the airborne route, some studies had investigated the presence of RSV RNA in aerosols with mixed results. While Chamseddine et al. (2021) could not detect RSV in aerosol samples collected near RSV-infected patients,^19^ several studies did.^20–25^ In line with them, Kulkarni et al. (2016) demonstrated that infants with RSV bronchiolitis, whether nursed in open wards or ventilated in intensive care, produce large numbers of aerosol particles containing infectious RSV. Importantly, many of these particles were smaller than 5 µm, capable of remaining airborne for prolonged periods and infecting human ciliated epithelial cells in vitro, thus providing support for the potential of aerosol spread.^22^ However, other than these limited reports, little information is available about the presence of RSV in aerosols.

Detection and quantification of viruses in aerosols could offer the opportunity to develop environmental surveillance systems, with potential application as early warning systems, detection of the virus in environments with limited clinical surveillance, and noninvasive monitoring of viral circulation in the environment.^26,27^

The aim of this study was to assess the suitability of long-term environmental surveillance of RSV infections based on aerosol samples. In line with this purpose, the main objective was to evaluate the association between the presence and abundance of RSV in aerosol samples collected during a long-term surveillance in a pediatric emergency corridor of a reference hospital in Eastern Spain and the incidence of RSV infections among the hospital’s pediatric population, given their high susceptibility to RSV infection.

## Methods

### Air sampling

A total of 200 consecutive aerosol samples were collected onto sterile 47mm quartz filters at 2.3 m^3^/h in the pediatric emergency corridor of the General University Hospital of Castelló de la Plana (Castellón, Spain) between the 3rd of January 2022, and the 16th of January 2023 (54 weeks). These samples were collected for 24-h on weekdays (Monday to Friday), with an additional 72-h sample collected over the weekend. No sampling was conducted during university’s vacation closure in August. Details of the sampling procedures, air sampling device specifications, sampling location, distance between patient cubicles and sampling equipment, and preventive measures are provided in the Supplementary Material.

### Viral RNA extraction and gene target quantification

Viral RNA was extracted from the filters following a similar procedure developed in our laboratory^28–30^ and detailed in the Supplementary Material. In summary, filters were inoculated with Mengovirus (MgV) as internal control to assess the efficiency of the genetic material extraction. Then, RNA was extracted using NucleoSpin® RNA kit (MACHEREY-NAGEL) to a final eluate of 60 µL that was analyzed in the RT-qPCR. The One Step PrimeScript™ RT-PCR kit (Takara, USA) was used for the detection and quantification of RSV genetic material using RSV primer of its matrix gene (M)^31^ (Table S1 and S2 in the Supplementary Material). Genomic RNA from human Respiratory Syncytial Virus A2 (ATCC VR-1540) was used as positive control to generate calibration curves for quantifying the RSV genetic material. RNase-free water was used as a negative control. The limits of detection (LoD) and limits of quantification (LoQ) were obtained according to method.^32^

### Epidemiological data

The hospital provided data on the daily pediatric emergency cases related with RSV infection covering the selected period of sampling. RSV cases in the Valencian Community^33^ and across Spain^34,35^ were also retrieved for the study period from official sources.

### Statistical analysis

Data of the RSV RNA concentrations in aerosols and daily RSV cases in pediatric emergency at the hospital were described using the median and the interquartile range (IQR) and range of concentrations (minimum and maximum). The χ^2^ test was used to evaluate the frequency of positive samples according to the sampling months and the Mann–Whitney test for the association between the number of positive filters and the number of RSV cases in their distribution by months.

A point-biserial correlation analysis was conducted to explore the relationship between the number of RSV cases in pediatric emergency departments and the positivity of air samples and the point-biserial correlation coefficient (*rpb*) was reported. For that analysis, each week was classified into two groups: positive or negative air sample.

Finally, Spearman correlation test was used to examine the relationship between RSV RNA concentrations and the RSV cases in pediatric emergency recorded in the hospital. Statistical tests were considered significant when the p-value was less than 0.05. All statistical analyses were conducted using R.

## Results

### Positivity of RSV in air samples

**Figure 1.**
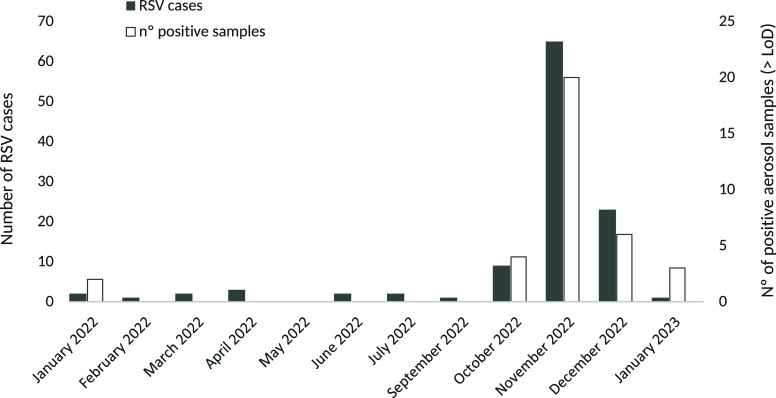
Number of RSV cases in pediatric emergencies and the number of positive aerosol samples for RSV, by month of sampling.

The average recovery of the internal standard (MgV) was 24.9 ± 23.7%. The RSV RNA has been detected in 35 samples (17.5%), with a median concentration (IQR) of 1.8 (4.1) gc/m^3^ (Table 1). RSV RNA was detected in air samples in 13 weeks out of the 54 sampled weeks, corresponding with the highest registration period of RSV cases in the pediatric emergency area at the hospital. The median (IQR) was 6 (10) RSV hospital pediatric cases during weeks with RSV RNA airborne samples detection and 0 (1) RSV cases for weeks without detection. The number of positive filters and RSV infection cases in the hospital followed a similar monthly distribution (*p*-value < 0,05) (Figure 1). November recorded the highest frequency of detection of RSV RNA in air samples (95.2%) and the maximum pediatric emergencies cases (65). RSV cases in the pediatric emergency department followed the same seasonal pattern observed in the Valencian Community^33^ and across Spain^34,35^ during the study period, with a marked peak between November 2022 and January 2023.

**Table 1.** Monthly distribution of RSV cases, number of air samples collected, proportion of positive samples, and RSV RNA concentrations (gc/m^3^; median, IQR, and range)

Month	RSV cases in hospital	Nº samples collected	Nº samples with RSV detection (Number of samples from weekend)	Detection frequency (%)	RSV concentrations median (IQR) (gc/m3), n^*^	Range of concentration RSV (gc/m3)
**January 2022**	2	19	2	10.5	0.89 (0.04), 2	0.86–0.93
**February 2022**	1	19	0	0.0	N.D.	N.D.
**March 2022**	2	19	0	0.0	N.D.	N.D.
**April 2022**	3	15	0	0.0	N.D.	N.D.
**May 2022**	0	20	0	0.0	N.D.	N.D.
**June 2022**	2	16	0	0.0	N.D.	N.D.
**July 2022**	2	20	0	0.0	N.D.	N.D.
**August 2022**	3	0	N.A.	N.A.	N.A.	N.A.
**September 2022**	1	10	0	0.0	N.D.	N.D.
**October 2022**	9	15	4 (1)	26.7	1.4 (0.84), 4	1.0–3.2
**November 2022**	65	21	20 (4)	95.2	4.2 (3.8), 20	1.3–9.5
**December 2022**	23	16	6 (3)	37.5	1.0 (0.14), 6	0.81–2.9
**January 2023** ^**^	1^**^	10	3	30	0.90 (0.12), 3	0.84–1.1
**Total**	**114**	**200**	**35 (8)**	**17.5**	**1.8 (4.1), 35**	**0.81–9.5**

* n is the number of samples with quantification.

** Counting until the end of the sampling, January 16.

N.D. is Non-Detected.

N.A. is Not Applicable.

In contrast, RSV RNA was not detected in any of the air samples collected between February and September, corresponding with the lowest incidence of RSV infection cases (Table 1), and in accordance with regional and national epidemiological data.^33–35^ The chronological sequence of RSV RNA detection in aerosol filters is shown in Figure S3 (Supplementary Material).

A positive correlation has been obtained between the RSV cases registered in the pediatric emergency area and the number of positive samples for RSV grouped according to sampling week (*ρ* = 0.62; *p*-value < 0.05) (Figure S4 in the Supplementary Material). Likewise, the point-biserial correlation analysis revealed a significant association between the number of RSV cases in the hospital’s pediatric emergency area and weeks with at least one positive air sample compared to weeks without positive samples (rpb = 0.63; *p*-value < 0.05) (Figure S5 in the Supplementary Material).

Over the sampling period, 35 filters tested positive. Among these, 24 cases corresponded with samples collected on sampling periods where RSV cases were reported at the hospital (*N* = 42), whereas 11 filters tested positive on days that no RSV cases were recorded at the hospital. There were also 18 days with at least one case of RSV at the hospital emergencies, but the filters tested negative or below the limit of detection.

### Quantification of RSV genetic material in air samples

**Figure 2.**
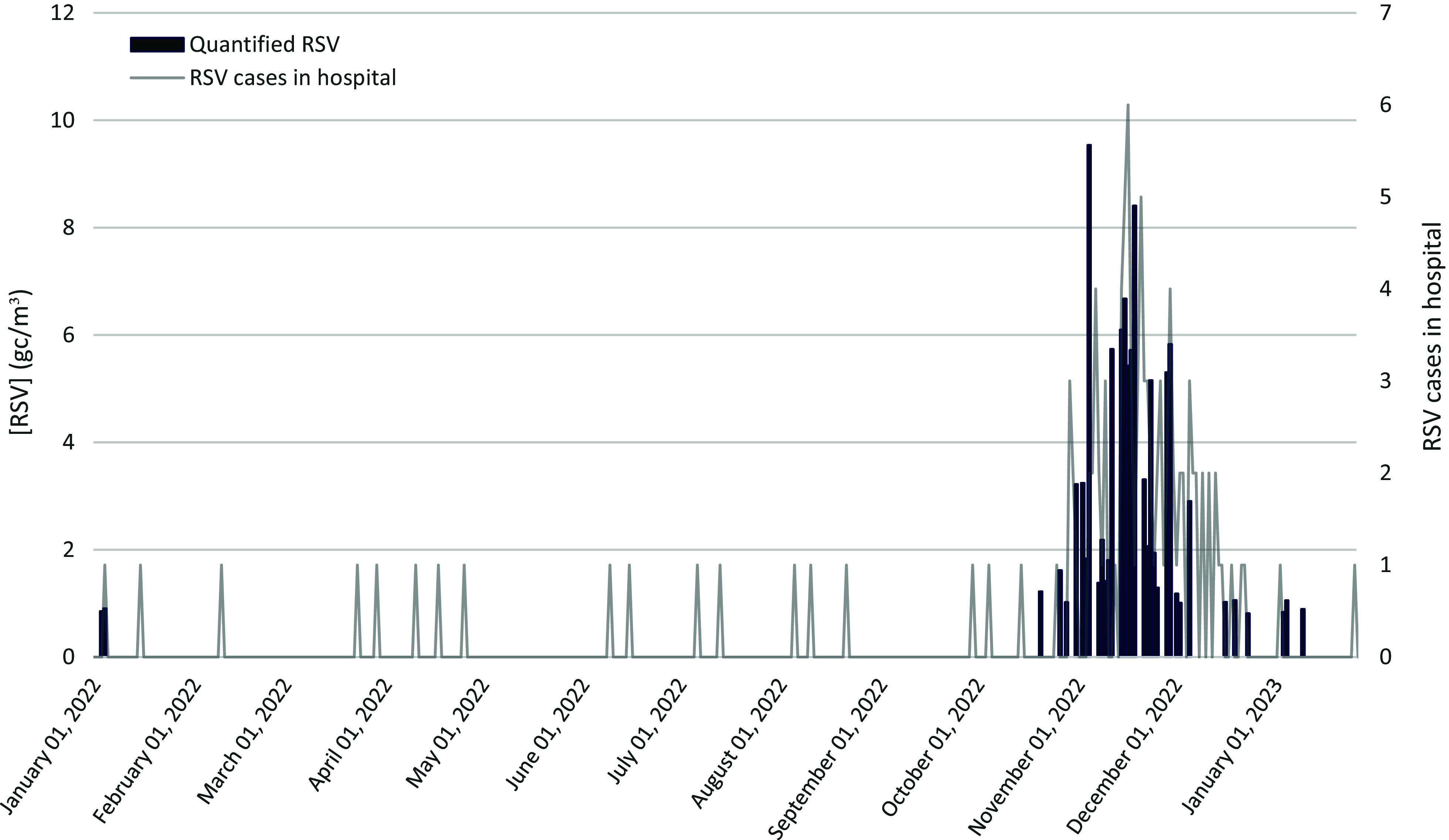
Assessment of RSV RNA concentrations in the pediatric emergency corridor and corresponding RSV cases in the pediatric emergency setting (03/01/2022 to 16/01/2023).

RSV genetic material was quantified in 35 air samples, with concentrations ranging from 0.81 to 9.5 gc/m^3^ (Table 1). Significant differences were observed in the medians of RSV concentrations obtained for each month of sampling (*p*-value < 0.05). November 2022 also had the highest monthly median RSV RNA concentration in the filters (4.2 gc/m^3^, Table 1). The presence of more than 1 confirmed case of RSV in the pediatric emergency room was required to detect and quantify RSV RNA in the pediatric emergency room in most of the cases (Figure 2).

A positive correlation was observed between the RSV cases registered in the hospital and the RSV RNA concentrations measured from aerosol samples (*ρ* = 0.55; *p*-value < 0.05) (Figure S6 in the Supplementary Material).

## Discussion

The presence and abundance of airborne RSV RNA collected at a hospital pediatric emergency department correlated with the number of RSV infections cases reported in the pediatric emergency department at the same hospital. These results do not constitute direct evidence of transmission, but highlight the need to further evaluate the role of aerosols in RSV spread. On the other hand, the results suggest that characterizing RSV RNA in aerosol samples could be useful for environmental surveillance applications.

### Detection of RSV RNA in air samples

The positivity rate of RSV RNA detection in the samples collected over the yearlong sampling period (17.5%) is similar to rates reported in other studies, that span 15% to 32% in various hospital spaces near RSV-infected patients.^20,21,25^ In addition, several studies have reported even lower positivity rate than in the present study spanning 1% in non-hospital settings^36^ to 3.8% in pediatric hospital rooms.^24^ Moreover, in two other studies, no RSV genetic material was detected in any of the samples analyzed^37^ including a study conducted in rooms with RSV-infected patients.^19^ On the other hand, certain studies have reported notably higher positivity rates, almost 100% in filters collected in rooms with RSV-infected children.^22^ This is similar to the high positivity rate observed in our study in November 2022 (95.2%), the month with the highest number of RSV pediatric cases and RSV RNA detection in filters.

The variability observed on the positivity rate in different studies might reflect differences in the environmental settings sampled. These might include the distance of the infected patient from the filter sampler, number of infected patients in the room and use of protective masks in infected patients. The location of the air sampling device closer to RSV-infected patients is likely to increase both detection rates and measured concentrations, as viral aerosols are more concentrated near the source before dilution and dispersion occur through ventilation and air mixing. Several studies conducted air sampling in close proximity to RSV-infected individuals,^20,22,25^ placing samplers at distances ranging from as little as 0.1 m^20^ to as far as 10 m^22^. In contrast, in our study the distances between patient rooms ranged from 4 to 17 m. The use of face masks by patients can substantially reduce the release of RSV-containing particles into the surrounding air, thereby lowering both the likelihood of detection and the measured concentrations in aerosol samples. Although mask use was mandatory throughout the study period, some children occasionally did not wear them. Nevertheless, most of the published studies did not provide information on whether patients were masked.^20,22,25^ It can also be associated with different analytical methods, such as the collection, filter storage and RNA extraction procedures, primers and target genes used or reverse transcription-quantitative polymerase chain reaction (RT-qPCR) cycling conditions. These parameters have been shown to affect detection rates from aerosol samples in other viruses, such as SARS-CoV-2.^29^

In all the aforementioned studies, bar three,^21,36,37^ sampling was conducted in hospital rooms with confirmed RSV-infected individuals. On the contrary, the samples from the current study were collected at the corridor of the Emergency Pediatric Unit without prior information of the presence or number of RSV cases at the location. Therefore, the analyst was blinded to the number of RSV infection cases attended at the Emergency Pediatric Unit. The proximity to infected individuals increases the likelihood of virus detection, as observed in Kulkarni and colleagues (2016), compared to studies where sampling is performed at a distance from RSV-infected persons.^37^ In the current study, it is highly probable that infected RSV individuals might have been near the sampling area at some point, since the air sampler was positioned in the pediatric emergency department hallway of the hospital, in a location likely capturing a large flow of patients and personnel.

Although some studies have reported concentrations of RSV RNA in aerosols in examination rooms, waiting areas and hallways, the lack of standardized methods makes direct numerical comparisons difficult.

### Limitations and strengths

This study has some limitations, particularly the uncertainty regarding the viability and infectious potential of the RSV detected in the air samples. While the presence of RSV RNA was confirmed, the lack of data on the survival and viral infectivity in aerosolized particles limits our ability to draw definitive conclusions about the role of aerosols in transmission. In this regard, only one study has provided evidence supporting RSV’s capability to remain infectious in the air within hospital environments.^22^ Another limitation stems from the absence of information regarding the total number of healthy or RSV-infected individuals passing through the sampling point each day, encompassing both healthcare staff and patients. In fact, only information of those cases diagnosed with RSV infection by the medical doctor was available and therefore included in the analysis. The absence of data due to sampling gaps (eg on public holidays or annual leave) is another constraint of the study. Nonetheless, the high percentage of temporal coverage allowed to assess the temporal trend of RSV RNA distribution along the sampling period.

Regarding the strengths of the study, to our knowledge, this study represents the first instance where RSV has been systematically monitored at a singular sampling point over the course of a year. Moreover, the site selected, the corridor of the pediatric emergency department of a reference hospital, is a location with a high probability to be frequented by vulnerable population infected with the RSV, such as children, infants, and babies. Hence, maximizing the probability to detect RSV genetic material extracted from the filters if the virus was airborne, as supported by the current results. This approach allowed a comprehensive analysis of RSV’s genetic load evolution and seasonal patterns in aerosols collected within a hospital environment.

This study has conducted a long-term surveillance of aerosols within a healthcare facility and compared the presence of airborne RSV RNA extracted from filter samples with the incidence of pediatric RSV infection cases in the reference hospital under study. The results show that both the presence and concentrations of airborne RSV genetic material follow a similar temporal pattern than the number of emergency pediatric RSV infection cases at the hospital over the course of the sampled year. Future studies should also explore the approach of using viral RNA detection in airborne samples for environmental surveillance in other community settings such as schools, shopping centers, or nursing homes.

RSV RNA was identified in aerosol samples collected within a healthcare environment. Although the presence of airborne RSV RNA correlates with pediatric infection cases, this does not by itself support aerosol transmission. Further research is needed to investigate the viability, infectious potential and transmission dynamics of airborne RSV in aerosols. Such research is crucial for enhancing our understanding of the role of aerosols in RSV transmission and informing evidence-based prevention strategies. On the other hand, the observed correlation between RSV RNA and pediatric infection cases suggests that environmental surveillance applications could be potentially developed using environmental aerosol sampling in strategic locations.

## Supporting information

Alfaro-Perez et al. supplementary material Alfaro-Perez et al. supplementary material

## Supplementary material

The supplementary material for this article can be found at https://doi.org/10.1017/ice.2025.10372.
