# Supplementary material for: Pediatric respiratory syncytial virus infections associated with hospital airborne viral genetic load detection
Source: Infect Control Hosp Epidemiol. 2025 Dec 17;47(3):284–9. doi: 10.1017/ice.2025.10372 (PMC12932919; doi:10.1017/ice.2025.10372)
Supplement: Alfaro-Perez et al. supplementary material [file S0899823X25103723sup001.docx]

**Paediatric respiratory syncytial virus infections associated with hospital airborne viral genetic load detection**

**Supplementary material**

C Alfaro^1*^, R de Llanos^1*^, LA Herrero Cucó^2^, & JM Delgado-Saborit^1,3#^

^1^ Department of Medicine, Faculty of Health Sciences. Universitat Jaume I, Avenida de Vicent Sos Baynat s/n, 12071 Castellón de la Plana, Spain

^2^ Hospital General Universitario Castellón, Castelló de la Plana, Spain

^3^ Epidemiology and Environmental Health Joint Research Unit, Foundation for the Promotion of Health and Biomedical Research in the Valencian Region, FISABIO-Public Health, FISABIO–Universitat Jaume I–Universitat de València, Av. Catalunya 21, 46020 Valencia, Spain

* These authors contributed equally to this work

# Corresponding author

Dr Juana Maria Delgado-Saborit, [delgado@uji.es](mailto:delgado@uji.es)

Perinatal Epidemiology, Environmental Health and Clinical Research. Department of Medicine, Faculty of Health Sciences. Universitat Jaume I, Avenida de Vicent Sos Baynat s/n, 12071 Castellón de la Plana, Spain

***Table of Contents:***

**Methods**

**Table S1.** Primers and probes used for RT-qPCR analysis.

**Table S2.** PCR Reaction Mix used in RT-qPCR analysis.

**Figure S1.** Scheme of the sampled corridor layout. The red star represents the location of the air sampler equipment.

**Figure S2.** Images of the paediatric emergency corridor. On the left, a view of the paediatric emergency corridor. On the right, a view of the sampling equipment.

**Results**

**Figure S3.** Temporal distribution of RSV RNA detection in a hospital corridor of paediatric emergency.

**Figure S4.** Correlation between the number of positives samples and RSV cases in paediatric emergencies according to the sampling week.

**Figure S5.** The results concerning the relationship between the weekly number of RSV cases and the samples are depicted in the following box plots.

**Figure S6.** Correlation between the concentrations obtained and RSV cases in paediatric emergencies.

# Methods

## Air sampling

The aerosol samples were collected using sterile 47mm quartz filters (Merck Millipore, Cork, Ireland; Ref.AQFA04700) positioned within a PM_2.5_ single-stage impactor, connected to an active air sampler (Derenda, J. Aguirre). The sampler operated at a flow rate of 2.3 m^3^/h. The air sampling was placed in the middle of a 27-meter paediatric emergency corridor, at a distance ranging from 4 meters from the nearest patient room to 17 meters from the farthest one. On one side of the corridor, there were 7 boxes (examination rooms), while on the other side, there was a waiting room, two observation rooms with multiple stations, and a vital signs room (Figure S1 and S2 in the Supplementary Material). The air inlet of the sampling head was positioned 1 meter above the ground level.

***Figure S1.*** *Scheme of the sampled corridor layout. The red star represents the location of the air sampler equipment.*


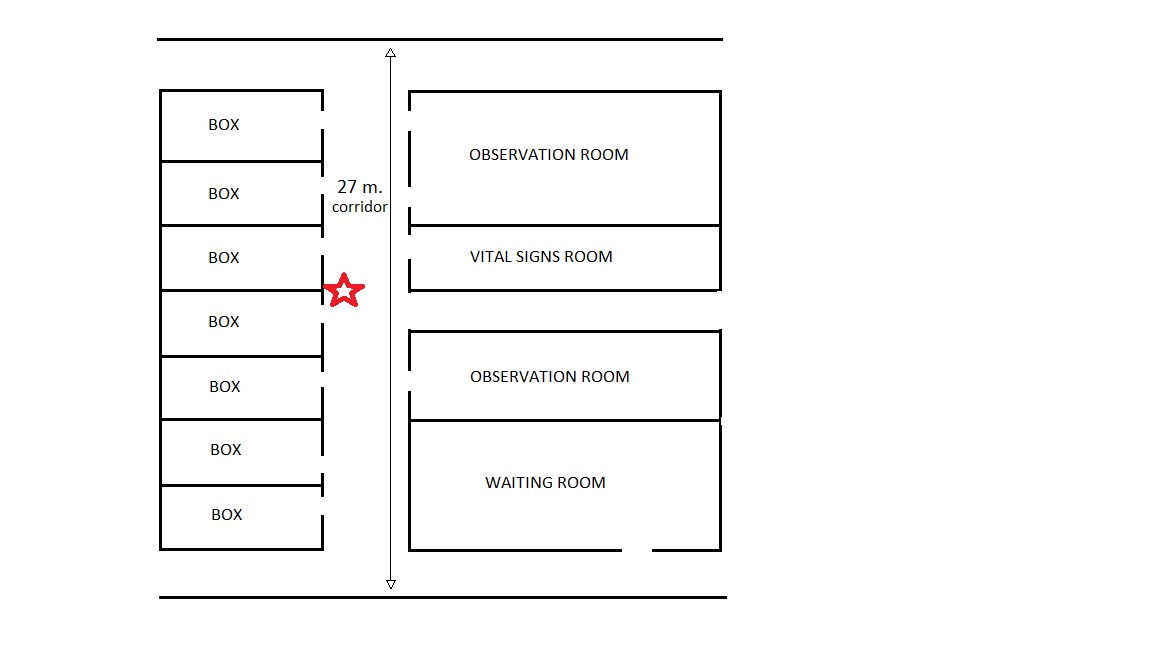


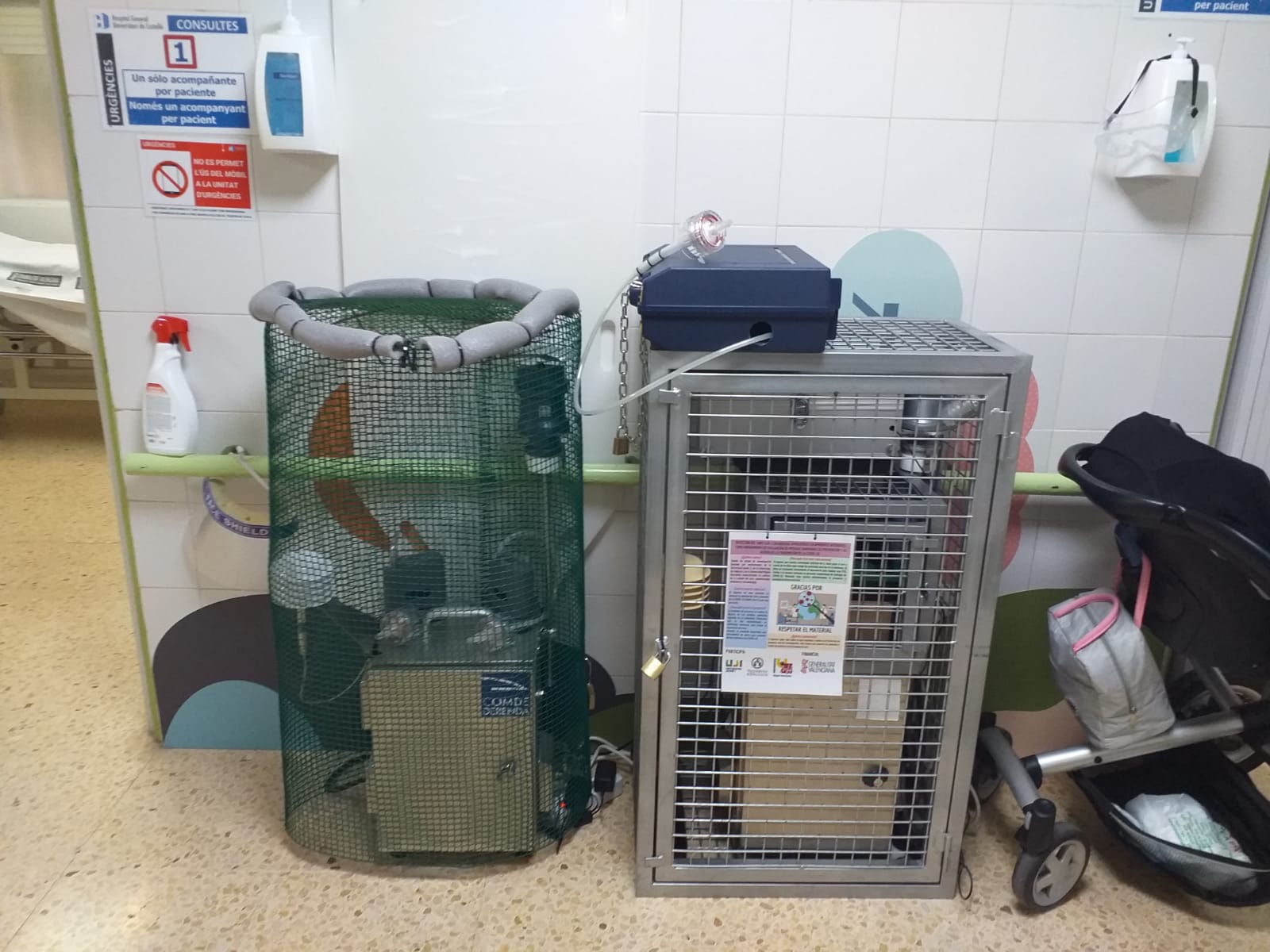

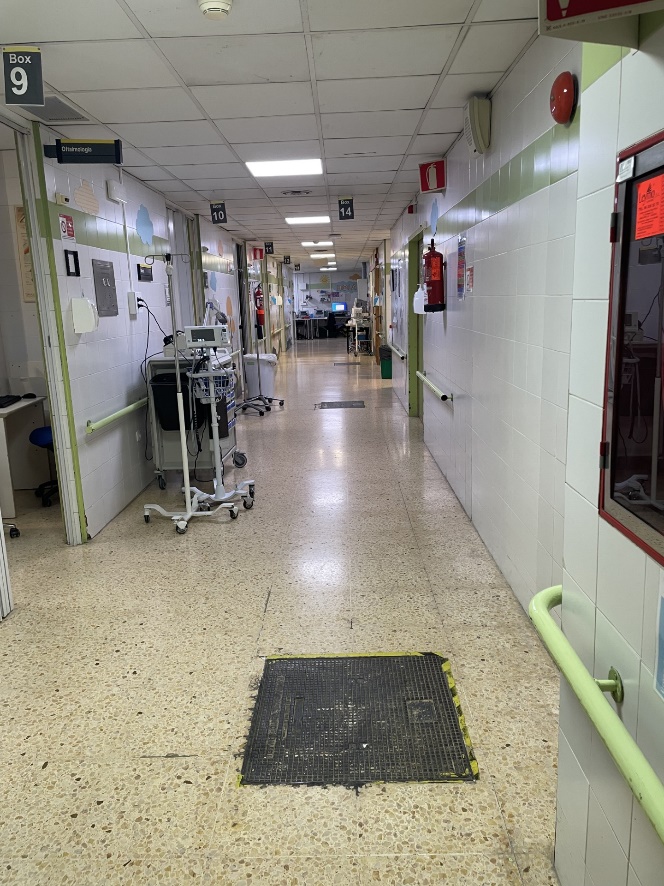
***Figure S2.*** *Images of the paediatric emergency corridor. On the left, a view of the paediatric emergency corridor. On the right, a view of the sampling equipment.*

##

## Paediatric emergency department: preventative measures

Universal masking (for healthcare workers, patients, and caregivers), together with the use of gloves and gowns, was mandatory during the study period, and all care areas were disinfected between patients. In waiting areas, physical distancing and occupancy limits were maintained whenever possible. Corridors functioned solely as passageways and were not used for patient observation or waiting.

Ventilation of patient cubicles was provided by a centralized HVAC (heating, ventilation, and air conditioning) system that also supplied the corridors, resulting in shared airflow between rooms and hallways. Natural ventilation was occasionally applied by opening windows in cases of suspected respiratory infection. The HVAC unit supplied 13.750 m³/h, resulting in 10 air changes per hour. The HVAC system consisted of a primary unit and several air handling units, all equipped with PSB-290 type filters with an 85% efficiency.

## Viral RNA isolation and gene target quantification

Viral RNA was extracted from the 47 mm quartz filters using the NucleoSpin® RNA kit (MACHEREY-NAGEL) following the procedure developed in our laboratory ^1-3^. In summary, the first step entailed the inoculation of 500 infectious units of Mengovirus vMC0 (CECT 100000) (MgV) into each sampled filter within a Class II safety cabinet as an internal control to assess the efficiency of the extraction. Then, each of the filters was rolled inward and placed in a 5 mL tube. Next, 1 g of 4 mm diameter glass beads and 0.9 mL of the extraction kit's lysis buffer were added, and the mixture was vortexed for 20 seconds. Subsequently, the 5 mL tube was inverted, and two holes were pinned in the cap inside a 50 mL tube and centrifuged at 8 500 rpm for 12 minutes. The collected volume eluate was processed according to the extraction kit's instructions. A final eluate of 60 µL in two filtrations was preserved in a 1.5 ml Eppendorf tube and stored at -80ºC until further analysis by RT-qPCR.

The One Step PrimeScript™ RT-PCR kit (Takara, USA) was used for the detection and quantification of RSV genetic material. Genomic RNA from human Respiratory Syncytial Virus A2 (ATCC VR-1540) was used as positive control to generate calibration curves for quantifying the RSV genetic material. RNase-free water was used as a negative control. The RSV primers were obtained from the literature and correspond to the matrix gene (M) ^4^ (Table S1 and S2). The RT-qPCR cycling program consisted of 10 min at 48 °C, 5 min at 95 °C, and 45 cycles of 15 s at 95 °C and 1 min at 55 °C. The RT-qPCR data analysis was performed using StepOne™ Software v2.3 analysis software.

Each sample was analysed in duplicate, and a sample was considered positive when at least one of the replicates had a Ct value below 40. Ct values above 40 were considered negative. The concentration of each sample was determined in gc/m^3^. The Ct values indicating the presence of RSV RNA were converted to genomic copies (gc) using the calibration curve, and the concentration in gc/m^3^ was calculated based on the sampled air volume. An arithmetic mean was then calculated from the duplicate positive values.

Finally, the limits of detection (LoD) and limits of quantification (LoQ) were obtained using a calibration curve performed in quintuplicate, following the methodology described by Forootan and colleagues (2017) ^5^. The LoD and LoQ for RSV were both 0.55 gc/m^3^.

***Table S1.*** *Primers and probes used for RT-qPCR analysis. The primers were synthesized by IDT (Integrated DNA Technologies, IDT).*

| Name | Description | Primer Sequence (5’>3’) |
| --- | --- | --- |
| RSV-F | RSV  Forward Primer | GGCAAATATGGAAACATACGTGAA |
| RSV-R | RSV  Reverse Primer | TCTTTTTCTAGGACATTGTAYTGAACAG |
| RSV-P | RSV  Probe | /56-FAM/CTGTGTATGTGGAGCCTTCGTGAAGCT/3BHQ_1/ |
| Mengo 110-F | Mengovirus  Forward Primer | GCG GGT CCT GCC GAA AGT |
| Mengo 209-R | Mengovirus  Reverse Primer | GAA GTA ACA TAT AGA CAG ACG CAC AC |
| Mengo 147-P | Mengovirus  Probe | FAM-ATC ACA TTA CTG GCC GAA GC-MGBNFQ |

***Table S2.*** *PCR Reaction Mix used in RT-qPCR analysis.*

|  | RSV | Internal control |
| --- | --- | --- |
|  | **RSV** | **Mengovirus** |
| RNase Free dH_2_O | 1.1 µL | 0.85 µL |
| 2X One Step RT-PCR Buffer | 5 µL | 5 µL |
| TaKaRa Ex Taq HS (5U/μl) | 0.2 µL | 0.2 µL |
| PrimeScript RT enzyme Mix II | 0.2 µL | 0.2 µL |
| Forward Primer | 0.5 µL ^a^ | 0.75 µL ^c^ |
| Reverse Primer |  |  |
| Probe |  |  |
| Extracted sample RNA | 3 µL | 3 µL |

^a^ Forward Primer (500 nM), reverse primer (500 nM) and probe (250 nM)**, described in table S1**

^b^ Primers (6.7 µM) and probe (425 nM), described in table S1.

**Results**

|  |  | 1 | 2 | 3 | 4 | 5 | 6 | 7 | 8 | 9 | 10 | 11 | 12 | 13 | 14 | 15 | 16 | 17 | 18 | 19 | 20 | 21 | 22 | 23 | 24 | 25 | 26 | 27 | 28 | 29 | 30 | 31 |
| --- | --- | --- | --- | --- | --- | --- | --- | --- | --- | --- | --- | --- | --- | --- | --- | --- | --- | --- | --- | --- | --- | --- | --- | --- | --- | --- | --- | --- | --- | --- | --- | --- |
| January 2022 |  |  |  |  | 1 |  |  |  | | |  |  |  |  | 1 | | |  |  |  |  |  | | |  |  |  |  |  | | |  |
| February 2022 |  |  |  |  |  | | |  |  | 1 |  |  | | |  |  |  |  |  | | |  |  |  |  |  | | |  |  |  |  |
| March 2022 |  |  |  |  |  | | |  |  |  |  |  | | |  |  |  |  |  |  |  |  |  | 1 |  |  | | |  | 1 |  |  |
| April 2022 |  |  |  |  |  |  |  |  | 1 | | |  |  |  |  |  |  | 1 |  |  |  |  |  | | | 1 |  |  |  |  |  |  |
| May 2022 |  |  |  |  |  |  |  | | |  |  |  |  |  | | |  |  |  |  |  | | |  |  |  |  |  | | |  | # |
| June 2022 |  |  | |  | | |  |  |  | 1 |  | | |  |  | 1 |  |  | | |  |  |  |  |  |  |  |  |  |  |  |  |
| July 2022 |  |  | | |  |  | 1 |  |  | | |  |  | 1 |  |  | | |  |  |  |  |  | | |  |  |  |  |  | | |
| August 2022 |  |  |  |  |  | 1 |  |  |  |  | 1 |  |  |  |  |  |  |  |  |  |  | 1 |  |  |  |  |  |  |  |  |  |  |
| September 2022 |  |  |  |  |  |  |  |  |  |  | | |  |  |  |  | | | |  |  |  |  |  |  |  |  |  |  | 1 |  |  |
| October 2022 |  |  |  |  | 1 |  |  |  | | |  |  | |  | 1 | | |  |  |  |  |  | | |  | 1 |  |  | 3 2 | | | 1* |
| November 2022 |  |  |  |  | 2 2 4 | | | 2 | 1 | 3 |  | 1 | | | 4 | 5 | 6 | 3 | 1 3 5 | | | 3 | 3 | 2 | 1 | 2 3 1 | | | 2 | 4 | 2 |  |
| December 2022 |  | 1 | 2 2 | | | 3 | 2 | 2 |  | 2 |  | 2 |  | 2 | 1 | 1 | 1 | | |  |  | 1 | 1 |  | | |  |  |  |  |  |  |
| January 2023 |  | 1 |  |  |  |  |  | | |  |  |  |  |  | | |  |  |  |  |  |  |  |  |  |  |  |  |  |  |  |  |

|  | Positive |  |  | RSV detection < LoD (negative) |  |  | Negative |  |  | Not sampled | n | nº RSV paediatric case(s) |
| --- | --- | --- | --- | --- | --- | --- | --- | --- | --- | --- | --- | --- |

***Figure S3.*** *Chronological distribution of RSV RNA detection in a hospital corridor of paediatric emergency. Red, yellow, and grey shaded boxes represent RSV positive, RSV detection with a CT value below the limit of detection, and RSV negative air samples, respectively. Crossed squares represent days with no sampling. The Y-axis represents the different months of the sampling period, and the X-axis represents the days of the month. (# represents a filter collected during May 31^st^, June 1^st^ and 2^nd^, 72 h sampling; X* represents a filter collected during the 31^st^ of October and 1^st^ of November, 48 h sampling)*


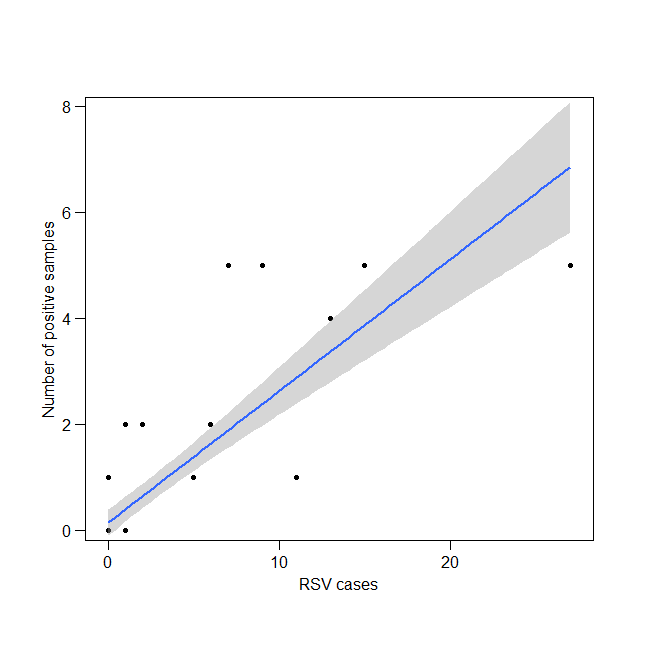


***Figure S4.*** *Correlation between the number of positives samples and RSV cases in paediatric emergencies according to the sampling week. Spearman's rho coefficient (**ρ) is* *0.62 (p-value<0.05). Linear regression shown with a blue line, and grey shadows represent the 95% confidence interval.*


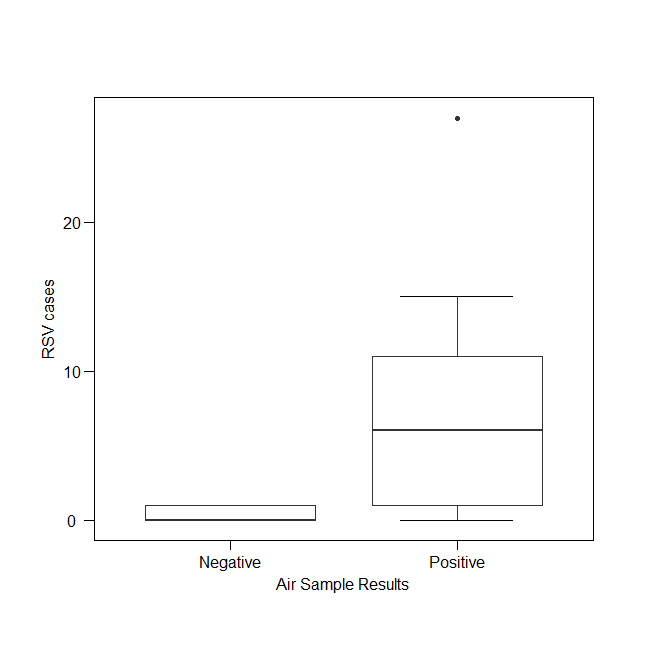


***Figure S5.*** *The results concerning the relationship between the weekly number of RSV cases and the samples are depicted in the following box plots. In these plots, the middle line signifies the median value, while the lower and upper hinges represent the 25th and 75th percentiles, respectively, and the points represent outliers (rpb=0,63; p-value<0,05).*


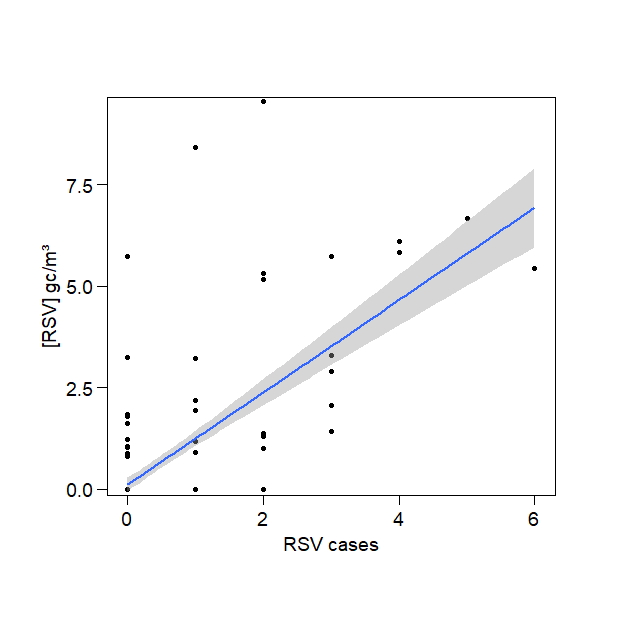


***Figure S6.*** *Correlation between the viral genetic concentrations obtained and RSV cases in paediatric emergencies. Spearman's rho coefficient (ρ) is 0.55 (p-value<0.05). The linear regression is shown with a blue line, and the grey shadows represent the 95% confidence interval.*

**References**

**1.** Barbera-Riera M, Porru S, Barneo-Munoz M, et al. Genetic Load of SARS-CoV-2 in Aerosols Collected in Operating Theaters. *APPLIED AND ENVIRONMENTAL MICROBIOLOGY* 2022;88.

**2.** Barberá-Riera M, Barneo-Muñoz M, Gascó-Laborda JC, et al. Detection of SARS-CoV-2 in aerosols in long term care facilities and other indoor spaces with known COVID-19 outbreaks. *Environmental Research* 2024.

**3.** Alfaro C, Porru S, Barberá-Riera M, et al. SARS-CoV-2 detection in aerosol from community indoor environments. *Building and Environment* 2024:111723.

**4.** Fry AM, Chittaganpitch M, Baggett HC, et al. The Burden of Hospitalized Lower Respiratory Tract Infection due to Respiratory Syncytial Virus in Rural Thailand. *PLOS ONE* 2010;5:e15098.

**5.** Forootan A, Sjöback R, Björkman J, Sjögreen B, Linz L, Kubista M. Methods to determine limit of detection and limit of quantification in quantitative real-time PCR (qPCR). *Biomolecular Detection and Quantification* 2017;12:1-6.
